# Eyes on the Fries: An Eye-Tracking Study of Motivated Attention and Calorie Labeling on Fast-Food Menus

**DOI:** 10.3390/nu18162620

**Published:** 2026-08-11

**Authors:** Rachel L. Bailey, Sun Young Park, Pooja Ichplani, Sol Lee

**Affiliations:** School of Communication, Florida State University, Tallahassee, FL 32306, USA

**Keywords:** visual food cues, calorie labeling, eye tracking

## Abstract

**Background/Objectives**: This study examines the effectiveness of calorie labeling on restaurant menus by investigating how visual attention is allocated between calorie information and food cues. Although calorie labeling policies are widely implemented, prior research suggests limited impact on reducing energy intake. **Methods**: Using eye-tracking technology, this study explored how menu design factors (specifically visual food cues) influence attention. **Results**: Results from a within-subject experiment (*N* = 82) indicated that calorie labels received significantly fewer visual fixations (in terms of frequency and duration) when food images were present. Conversely, calorie labels did not reduce attention to food cues, highlighting an asymmetry in attentional allocations. Interestingly, restricted eaters were associated with more frequent attention to calorie labels but not with longer sustained attention to those labels. **Conclusions**: Overall, findings suggest that the motivational responses elicited by food cues undermine the effectiveness of calorie labeling by diverting attention away from nutritional information, limiting its utility as a public health intervention.

## 1. Introduction

A federal requirement mandating that chain restaurants display calorie information on menus was passed as part of the Affordable Care Act. Although this policy is often framed as a public health success, data were available prior to it, ultimately going into effect in 2018 [1], suggesting that calorie labeling alone is inadequate in reducing energy intake and, therefore, has limited implications in reducing overweight and obesity incidence [2,3,4,5,6,7]. Despite these findings, a small body of work suggests that under some specific circumstances, calorie labeling may be more effective [8,9,10,11,12]. To better understand the mechanisms underlying decisions related to calorie labeling in food menus, this paper presents a study using eye-tracking technology to investigate how common fast-food menu design features may undermine the effectiveness of calorie labeling.

### 1.1. Calorie Labeling Effectiveness

Calorie labeling as a policy has its roots in the information deficit model, which posits that if individuals are provided important scientific knowledge, they will have more positive attitudes toward science, more trust in science, and more intentions to behave in ways recommended by scientists [13,14]. However, this perspective overlooks important considerations, including cultural and religious beliefs, reactance, social norms, motivations, and pre-existing attitudes and preferences, that play a critical role in predicting how people behave and, in this case, what they will eat and why. Consequently, it comes as no surprise that several reviews report no significant differences in energy intake associated with calorie labeling [3,5,6,7]. Critiques of the studies identified in these reviews have questioned their validity given various methodological limitations, but additional research attempting to address these and other concerns have also found little to no reduction in calories consumed [4,8,9,15].

Though most studies suggest that menu calorie labeling is generally ineffective for most people, some evidence suggests that calorie labeling can be effective under specific conditions. For example, calorie labeling in dine-in restaurants has been associated with reduced calorie selection [10]. Similarly, the context for calorie and nutrition labels and the design of menus have also been investigated. For instance, when calorie labels are paired with recommended daily intake information, individuals select fewer calories than when labels are presented alone [12]. Further, calorie labeling is more effective when positioned in accordance with reading patterns (i.e., left to right for English readers) [16], and the placement of calorie labels relative to price information affects consumer decisions as well [17,18]. Additionally, the juxtaposition of calorie labels with images of the food, the energy density of the food, and the number of items available for selection have been shown to affect calorie label effectiveness [8]. More specifically, images of food tended to increase the number of calories selected for intake, but if both higher- and lower-energy-density items were available, images of food only increased calorie selections when calorie labels were not present. When images of food and calorie labels were present in these larger and more varied arrays, slightly fewer calories were selected compared to calorie labels alone without images. This indicates that the images may have provided additional information that made the lower-energy-density items more motivating. This conclusion is bolstered by the finding that this specific type of menu (i.e., more items, greater energy density variety, and images present) elicited the greatest number of calories selected on average [19].

The underlying mechanisms explaining why these specific menu designs influence calorie label effectiveness are those that modulate visual attention. Because the human brain’s information processing capacity is limited [20], consumers facing a complex menu may not be motivated to evaluate every option and instead become highly selective about the information they encode [21,22]. Consequently, visual attention mechanisms act as gatekeepers, determining exactly which choice alternatives are included or excluded from a consumer’s consideration set [23,24]. Furthermore, meta-analytic eye-tracking data reveal that external visual factors (e.g., visual salience and positioning of information) play a role that is equal to or even larger than a consumer’s internal cognitive goals (e.g., health consciousness) in capturing this attention [25]. These findings suggest that the effects of calorie labeling depend not only on whether calorie information is present but also on how it is visually configured within the menu environment and how this configuration influences visual attention mechanisms.

Eye-tracking studies on the visual formatting of menus show that numeric, color-coded, and physical activity-based menu labeling can affect visual attention, format preferences, and healthier selections on fast-food menus, with physical activity-based labels being the most promising [26]. Calorie disclosure and color coding have also been examined with eye tracking for quick-service restaurant menus, supporting that these elements can influence gaze behavior and menu choices [27]. This suggests that calorie labels suffer from an attentional competition problem that requires a visual design intervention rather than from a problem of information availability.

A food or calorie label can only influence behavior if its physical design successfully competes for visual attention against the surrounding clutter of the food decision-making environment. Eye-tracking technology and an empirical investigation of visual fixations offers a useful way to examine visual attention mechanisms by showing how attention is allocated across competing menu features before selection occurs.

Building on these findings, the present study approached the issue from the perspective of attentional competition. To that end, we examined how the ubiquitous inclusion of images of foods, which are known to draw motivated attention, on menus with and without calorie labels altered the visual attention allocated (i.e., number of and total duration of visual fixations) to these different features of the menus.

### 1.2. Visual Food Cues and Motivated Attention

Without adequate metabolic and nutrient balance, organisms cannot meet the biological demands necessary for survival and reproduction. Consequently, food is a primary appetitive, motivationally relevant stimulus. Appetitive stimuli automatically activate motivational systems that guide attention and information processing [28,29,30]. The goal of this activation is to facilitate information intake in pursuit of biologically relevant opportunities [20]. Consistent with this framework, food stimuli are detected more quickly and accurately, are better remembered, and elicit stronger positive responses than less motivationally relevant stimuli [31,32,33,34,35,36]. Further, visual food cues are strong predictors of appetitive motivational system activation, even if they are paired with negative verbal cues [37]. In marketing contexts, such cues also increase selection likelihood and speed and can even inflate perceptions of healthfulness [31,32,38].

Importantly, certain types of food cues are more salient than others. Food cues rich in sensory detail trigger automatic appetitive responses, often without deliberate cognitive processing, because these cues signal that the foods afford edibility. These affordances and the automatic motivational responses they derive are particularly strong when cues most closely resemble real food and share perceptual features with actual food (e.g., appetizing visual depictions). Such cues may operate partly through taste-related sensory inferences, signaling qualities such as flavor intensity, richness, and freshness that increase hedonic appeal, positive affective responses, attentional engagement, recognition-based encoding, and food-related behavioral intentions [39,40,41].

Thus, visual food cues are motivationally relevant stimuli that capture attentional resources, but it may also be that they influence distinct types of attention differently. Selective attention refers to the process by which individuals prioritize certain stimuli for processing while filtering out competing information in the environment. Although selective attention can be guided by controlled, goal-directed processes, it is also highly influenced by automatic processes that rapidly orient attention toward salient, motivationally relevant, or reward-associated stimuli [30]. Contemporary models therefore conceptualize selective attention as emerging from the interaction of both controlled top-down processes and more automatic bottom-up and learned attentional biases, e.g., [20,42,43].

Deliberative attention, in contrast, refers to the controlled, effortful allocation of cognitive resources toward consciously evaluating, interpreting, and reflecting on information. Unlike more automatic forms of selective attention, deliberative attention is primarily goal-directed and relies on higher-order cognitive processes such as sustained focus, reasoning, and decision making. It is typically engaged when individuals carefully weigh information, consider long-term consequences, or intentionally regulate behavior [44,45].

With this general model of motivated visual attention in mind, we can consider more carefully why visual food cues on menus may influence calorie label effectiveness. First, visual food cues are going to automatically draw selective attention towards themselves, but calorie labels do not have this type of automatic motivated attention bias in their favor. Thus, from an automatic selective attention perspective, more visual attention should be allocated to the food cues. Second, when highly salient motivational cues automatically capture visual attentional resources, they may also reduce the attentional resources available to processing peripheral information such as calorie labels [20,46,47]. When appetitive activation is high, attentional processing narrows, potentially limiting the encoding of other less motivationally relevant information. This raises the possibility that realistic visual food cues may undermine the effectiveness of calorie labeling not only by attracting attention to themselves but also by diverting attention away from these labels.

Lastly, reward history related to food should be considered. Failing and Theeuwes [43] suggested, based on reviewed evidence, that selective attention cannot be explained solely by the dual processes of top-down (goal-directed) and bottom-up (stimulus-driven) attention. Instead, they proposed a third source of attentional control called selection history, which is the idea that past attentional experiences, especially those tied to reward, continue to bias attention even when they are no longer relevant to current goals. This implies that repeated exposure to visual food cues tied to rewarding experiences (e.g., eating high-fat, high-sugar food) may strengthen automatic attentional biases that then compete with any deliberative decision-making processes that might consider calorie labels.

Empirical evidence supports this attentional competition account. Kemps et al. [48] found that low-calorie-food cues reduced calorie consumption only when presented alone; when paired with high-calorie cues, this effect diminished. Similarly, Keegan et al. [49] demonstrated that the spatial separation of low- and high-calorie items increased selection of lower-calorie options. These findings indicate that the influence of calorie labeling depends not only on its presence but also on the broader attentional environment in which it is embedded.

Taken together, these theoretical propositions and empirical data suggest that when visual food cues are present, they attract attention automatically and may draw attentional resources away from calorie labels. Further, because food cues are more automatically attention grabbing (i.e., automatic selection) and also may be reward-salient due to their selection history [43], it is likely this effect may also be apparent in more deliberative processing. Only those individuals who are motivated in a more controlled and volitional way to use calorie labels may allocate enough attentional resources to them to use them effectively. Thus, restricted eaters (those who restrict types and amounts of food to control weight) are more likely than non-restricted eaters to attend to calorie labels. However, it is questionable whether the additional information provided by calorie labels draws any sustained attentional resources away from food cues compared to when calorie labels are absent, even for restricted eaters. Thus, we predict and ask:

**H1.**  *When visual food cues are present, calorie labels will receive (a) fewer fixations and (b) a shorter overall duration of fixations compared to when visual food cues are absent.*

**H2.**  *Restricted eaters will exhibit (a) more fixations and (b) a longer overall duration of fixations on calorie labels compared to non-restricted eaters.*

**RQ1.**  *Will the number and duration of fixations differ for food cues when calorie labels are present?*

These hypotheses and the research question were tested using a within-subject experimental design in which screen-based eye-tracking technology was used to track where individuals were looking on screen as they selected food items from simplified, digital fast-food menus.

## 2. Method

Participants (*N* = 93) were undergraduate students enrolled at a large public university in the southeastern United States who received course credit for participation. Data from two participants were excluded due to equipment or experimenter error. An additional 9 participants were removed due to missing data, resulting in a final sample of *N* = 82. The sample consisted primarily of young adults (M_age_ = 22.05, SD_age_ = 3.62), with a majority identifying as White (73.1%) and female (66.7%). Participants completed a 2 (calorie label: present vs. absent) × 2 (visual cue: present vs. absent) × 3 (menu type: lower energy density/6-item; higher energy density/6-item; mixed/12-item) × 4 (restaurant exemplars) mixed factorial experiment. This design required the creation of 48 simplified menus varying across these factors. Menus contained either 6 or 12 items, though this factor was not fully crossed with all others. Twelve-item menus included both higher- and lower-energy-density items drawn from the smaller arrays, whereas six-item menus contained only one energy density category. Including different types of food items creates different decision-making contexts. While we are interested in this as an IV, these data are not reported here. With this design, the inclusion of multiple menu types to examine visual food cue influences should be viewed as an effort to increase the generalizability of the findings to different types of menus.

Further, menu items were drawn from four fast-food restaurants (Arby’s, Burger King, Taco Bell, and Chick-fil-A) to enhance generalizability across food categories rather than focusing on a single type of item. This factor was also not fully crossed. Each participant viewed and made selections from 12 menus, representing one instance of each combination of calorie labeling, visual cues, and menu type. Participants were randomly assigned to select what they considered a typical meal from each menu, with each menu tied to only one of the four restaurants. They were informed that the study examined how menu characteristics influence decision making, and no deception was employed.

### 2.1. Independent Variables

**Figure 1:**
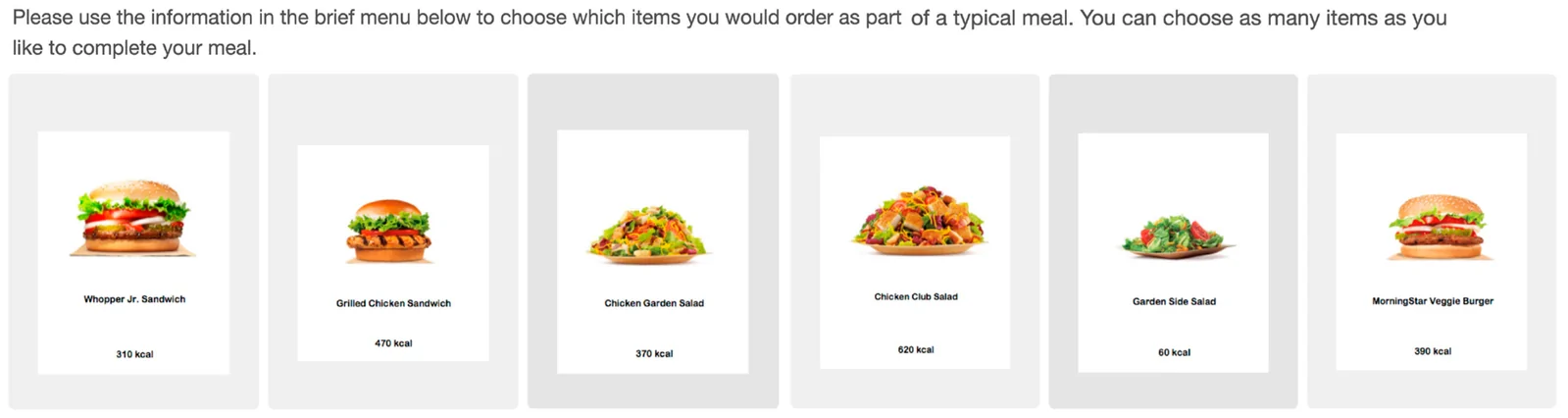
Example of lower-energy-density small array with calorie label and visual cue present.

**Calorie Labels**. Calorie information was either displayed beneath each menu item (e.g., “Whopper Sandwich, 660 kcal”) or omitted, leaving only the item name (e.g., “Whopper Sandwich”; see Figure 1).

**Visual Food Cues**. Images corresponding to each food item were obtained from the restaurants’ online menus. These images were either presented alongside the item name or not included.

**Arrays**. Menus varied in size (6 vs. 12 items) and energy density. Energy density (kcal/g) was calculated using nutritional information provided by the restaurants. Items were categorized as relatively higher or lower in energy density within each restaurant’s menu. Both categories included a mix of entrées and side items to maintain variety. Six-item menus contained only higher- or only lower-energy-density items, while 12-item menus combined both sets. Detailed information is presented in Table 1. Statistical analysis confirmed that lower-energy-density items were significantly different from higher-energy-density items (*F*(1,5) = 88.63, *p* < 0.001).

**Repetition**. Menu items were sampled from Arby’s, Burger King, Taco Bell, and Chick-fil-A to allow for generalization across types of fast food rather than focusing on a single type. These restaurants were selected due to their high levels of advertising [50] and their diverse offerings (e.g., sandwiches, burgers, chicken, tacos). These menus have been used in previous studies to examine calorie labeling effectiveness, albeit with different participants [8,19].

**Restricted Eating**. Individuals (*n* = 12) indicated in an open-ended question whether they restricted their intake of certain foods in any way (e.g., vegan, vegetarian, don’t eat red meat, limit carbohydrates). In analyses, 0 = no restriction and 1 = restriction.

### 2.2. Dependent Variables

**Automatic Selective Attention (Number of Fixations)**. Number of fixations was used to index automatic selective attention. Number of fixations is a metric that counts how many times a user’s gaze rests within a specific area of interest (AOI) across a specified length of time. A fixation is defined here as a sequence of raw gaze points where eye movement is relatively still, falling below a set velocity threshold using Tobii’s I-VT (Velocity Threshold Identification) filter. In other words, a fixation is defined as when the eye rests on something. Number of fixations was collected using a Tobii Pro Nano screen-based eye-tracking device (60 Hz sampling rate) attached to a 13-inch HP laptop in a lab-based setting with adequate lighting. Participants were seated approximately 2 feet from the laptop, which was situated on a desk. No chin rest was used; participants were able to move freely. Tobii Pro Lab software version 25.19 was used to analyze and calculate the number of fixations. AOIs were manually coded by trained research assistants.

**Controlled Deliberative Attention (Duration of Fixations)**. Total duration of fixations was used to index more controlled deliberative attention. Duration of fixations is a metric that sums all fixation times within an AOI during a specific time. In other words, duration of fixations is how long the eye rests on something. Duration of fixations was collected using a Tobii Pro Nano screen-based eye-tracking device attached to a 13-inch HP laptop. Tobii Pro Lab software was used to analyze and calculate the total duration (sum) of fixations (see above) within AOIs.

### 2.3. Covariates

**Hours Since Eating**. Individuals were asked to report how many hours ago they last ate something in an open-ended response. This was asked after they had viewed and selected from all menu questions. On average, individuals had eaten within 7 h of beginning the study (*M* = 6.62, *SD* = 5.91).

**Self-Reported Hunger**. Individuals were asked to respond on a 7-point scale from 1 (not at all) to 7 (extremely) how hungry they currently felt at the beginning of the study prior to seeing any menus. Individuals were not very hungry on average (*M* = 3.33, *SD* = 1.54).

**Body Mass Index (BMI)**. Individuals were asked to report their height (in pounds) and weight (in inches). BMI was computed using the following formula: BMI = (weight/height^2^) × 703. Individuals were in the ideal-BMI category on average (*M* = 23.47, *SD* = 3.96), though underweight and obese individuals were also present in the sample (range: 15.12 to 39.15).

**Perceived Current Stress**. Individuals were asked to respond to the ten-item PSS10 [51] to index their current level of perceived stress. Perceived stress in the sample was moderate and normally distributed (*M* = 2.85; *SD* = 0.58, *α* = 0.83).

**Race**. Individuals were asked to self-report their race/ethnicity, with the ability to choose multiple races when applicable. White/Caucasian was coded as 0, Black as 1, Asian as 2, Latino(a) as 3, and other or multiple as 4.

### 2.4. Procedure

Upon arrival at the lab, each participant was invited to read the informed consent and ask any questions. Next, participants were seated at a desk and provided a laptop. Participants were prepared with psychophysiological data collection sensors (these data are not reported here; see Supplementary Table S1). Next, participants’ eye tracking was calibrated using a 9-point calibration procedure. Then, participants viewed and made selections from 12 menus (see above). Both menu order and item order within menus were randomized. After completing the selection task, participants answered questions assessing eating habits, nutrition knowledge, and demographic characteristics. Upon completion of questions, sensors were removed from the participants, and they were thanked, debriefed, and dismissed. The entire procedure took approximately 30 min.

### 2.5. Data Analysis

To account for latent individual differences that standard Ordinary Least Squares (OLS) regressions often overlook, this study utilized multilevel modeling [52]. Specifically, a series of two-level random intercept models were applied to address the repeated-measures nature of the data. The level 1 unit of analysis consisted of each observation of the dependent variables across menu conditions, yielding 984 observations (*n* = 82 participants × 12 menu conditions = 984), and these observations were nested within participants at level 2 (*N* = 82). All models were estimated using HLM 8.0 software [53] by using restricted maximum likelihood estimation.

To determine the proportion of variance attributable to between-subject differences, null models were estimated for all eight experimental combinations (2 analysis contexts × 4 dependent variables). The intraclass correlation coefficients (ICCs) were calculated based on the within-subject and between-subject variance components produced by these models.

In the analysis context of calorie labels present vs. absent, the ICCs for food cue fixation frequency and fixation duration were 0.76 and 0.72, respectively. Conversely, the ICCs for calorie label fixation frequency and fixation duration were negligible (<0.001).

In the analysis context of food cue present vs. absent, the ICCs for calorie label fixation frequency and fixation duration were 0.44 and 0.35, respectively. However, the ICCs for food cue fixation frequency and fixation duration were again negligible (<0.001).

## 3. Results

### 3.1. Hypothesis 1

Hypothesis 1 predicted that when visual food cues were present, calorie labels would receive (a) fewer fixations and (b) a shorter overall duration of fixations compared to when visual food cues were absent. There was a significant mean difference in how participants allocated their visual attention between food cues and calorie labels across the different menu arrays across participants on average. Participants engaged in significantly fewer fixations on calorie labels when food images were present compared to when they were absent (*B* = −8.30, *S.E*. = 2.27, *p* < 0.001), meaning that when food images were added to the menu, fixations on calorie labels significantly decreased by 8.30 fixations on average. Similarly, the presence of food cues significantly reduced the duration of fixation to calorie labels (*B* = −4.36, *S.E*. = 1.07, *p* < 0.001), meaning that the presence of food images reduced the time spent looking at calorie labels by 4.36 s on average. Therefore, Hypothesis 1 was supported. Table 2 and Table 3 report the model results.

### 3.2. Hypothesis 2

Hypothesis 2 predicted that restricted eaters would exhibit (a) more fixations and (b) a longer overall duration of fixations on calorie labels compared to non-restricted eaters. Analyses revealed that being a restricted eater increased the number of fixations on calorie labels (*B* = 11.30, *S.E*. = 4.84, *p* < 0.02), meaning that restricted eaters exhibited 11.3 more fixations on calorie labels on average than non-restricted eaters. However, restricted eaters did not exhibit a longer duration of fixations on calorie labels (*B* = 3.44, *S.E.* = 2.11, *p* = 0.11) than non-restricted eaters. Hypothesis 2 was partially supported.

### 3.3. Research Question 1

**Figure 2:**
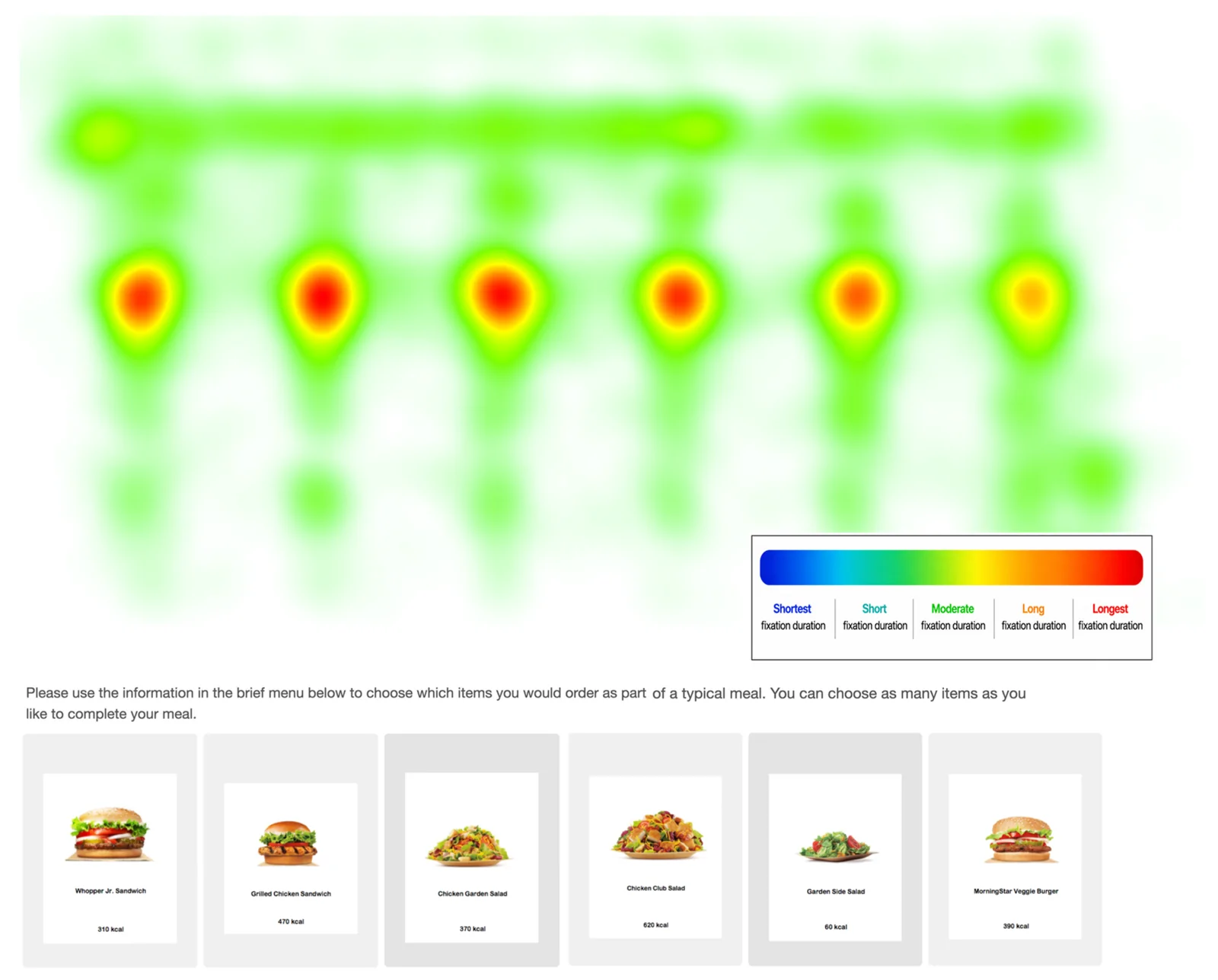
Small arrays of lower-energy-density items: (**top**) heat map pattern of fixation duration, and (**bottom**) exemplar for comparison.

Research Question 1 asked whether the number and duration of fixations would differ for food cues when calorie labels were present. Analyses revealed that the presence of calorie labels did not significantly alter the number of fixations on food cues compared to when calorie labels were absent (*B* = −0.28, *S.E.* = 2.55, *p* < 0.91). As a comparison, when food cues were present, individuals exhibited on average ~48 more fixations on these AOIs (*B* = 48.25, *S.E.* = 6.58, *p* < 0.001) compared to when they were absent. Additionally, analyses revealed that the presence of calorie labels did not elicit a significant difference in the overall duration of fixations on food cues compared to when calorie labels were absent (*B* = −0.20, *S.E*. = 0.85, *p* < 0.81). Again, as a comparison, when food cues were present, individuals spent on average ~13 more seconds on food cue AOIs compared to when they were absent (*B* = 13.03, *S.E*. = 1.99, *p* < 0.001). See Figure 2 for an example heat map depiction of the duration of fixation. Table 4 and Table 5 report the model results. Further, no individual differences were found for restricted vs. non-restricted eaters.

## 4. Discussion

The present study examined how visual attention to calorie labels is shaped by the presence of competing stimuli, namely, visual food cues. Using eye-tracking metrics, this research provides insight into how individuals allocate visual attentional resources in fast-food environments and offers implications for the effectiveness of calorie labeling as a public health strategy. In general, these findings add to the literature suggesting that calorie labels are of limited effectiveness [3,4,5,6,7], especially in contexts where more motivating information is competing for that attention [8,19].

Consistent with Hypothesis 1, calorie labels received significantly fewer fixations and shorter fixation durations when visual food cues were present. Further, findings related to Research Question 1 demonstrated that not only did food cues attract more fixations and longer durations than calorie labels, but the presence of calorie labels also did not significantly influence attention to food cues. The number and duration of fixations on food stimuli remained constant regardless of whether calorie information was present. This asymmetry is notable: while food cues reduced attention to calorie labels, calorie labels did not reduce attention to food cues. This is in line with the first two arguments for why calorie labels are ineffective in the presence of food cues—food cues garner automatic selection and reduce the attentional resources available to peripheral, less motivationally relevant information like calorie labels.

Examining the variance decomposition of the models adds nuance to this explanation. Attention to calorie information exhibited negligible between-person variability (ICCs ≈ 0.001), indicating that it was almost entirely stimulus-driven. When calorie labels were present, individuals looked at them in this experimental, decision-making context. In contrast, attention to food showed substantial between-person variance (ICCs ≈ 0.72–0.76), reflecting trait-like individual differences in attentional allocation. Motivational reactivity to food imagery was apparent; those who paid more visual attention to food cues did so consistently.

Practically, these findings suggest that, in the absence of visually motivating food stimuli, individuals are more likely to attend to informational elements such as calorie content; however, simply adding calorie information to typical fast-food menus that often include more appealing visual imagery may be insufficient to shift automatically motivated attentional priorities, especially for more motivationally reactive individuals. Food images, as primary appetitive stimuli, garner automatic attention and appear to suppress attention to less automatically motivating information in general. When these cues are removed, attentional resources may be redirected toward textual or numerical information, increasing engagement with calorie labels. This pattern aligns with models of visual attention that emphasize the prioritization of motivationally relevant stimuli over less relevant information, as discussed above. While calorie labels can attract some visual attention in simplified contexts, their effectiveness, when it relies on visual attentional selection only, is likely to be even more reduced in realistic environments where visually appealing food cues are present in abundance. Calorie information would need to be made more motivationally relevant than it is for most consumers, or the presentation of food cues would need to be modified to improve the impact of calorie labeling interventions from an attentional selection point of view. Of course, higher-order decision making and goals may intervene upon the types of motivated choices one makes in consumption contexts. Paying more attention to a calorie label does not guarantee the selection of fewer calories, but in order for the information provided in a calorie label to be useful in a decision-making context, it must be attended to. This is apparent in the data relevant to restricted eaters.

Consistent with Hypothesis 2, restricted eaters fixated more on calorie labels than non-restricted eaters, though they did not spend a longer amount of time overall on them. This is in line with previous empirical data suggesting that restrained eaters or individuals actively dieting may respond differently to food-related stimuli and calorie labeling [9]. Individuals may reduce intake following exposure to cues that activate dieting goals, such as lower-energy-density or health-oriented foods [48,54,55]. In general, this supports the notion that motivation related to controlled, volitional goal setting can encourage use of calorie labels; that is, if you are motivated to use them to further your own weight or health goals, you will.

However, the pattern of gaze behavior is interesting and suggests some support for the third argument for why calorie labels are ineffective in the presence of food cues. The reward history of fast-food decision making is likely also playing a role. As Failing and Theeuwes [43] suggest, selective attention cannot be explained solely by the dual interacting processes of goal-directed and stimulus-driven attention. They propose that past attentional experiences, especially those tied to reward, continue to bias attention even when they are no longer relevant to current goals. The repeated exposure to visual food cues tied to rewarding experiences, such as eating high-fat, high-sugar food in fast-food contexts after selecting from visuals, may strengthen automatic attentional biases that then compete with goal-directed, deliberative, decision-making processes, not just automatic selection processes.

Restricted eaters had a greater number of fixations on calorie labels compared to non-restricted eaters, but they did not spend more total time on calorie labels. This indicates that calorie labels were successful at competing for their visual attention but inadequate to sustain it. It may be that they switched back and forth between the calorie labels and other information more quickly. In this simplistic menu context, the only other information available was the name of the item, the food cues when present, the question text, or other information on screen not relevant to the decision (e.g., standard tool bars). Though it was not statistically significant, restricted eaters did fixate on food cues longer than non-restricted eaters, which would also support this explanation. They are motivated in a volitional way to attend to the calorie label but are continuously drawn back by the food cues. This is consistent with ideas of incentive salience, in which visual food cues acquire reward value and priority in the attentional system through repeated associations with consumption [56], as discussed above [43]. Neuroimaging research indicates that food images activate reward-related neural regions, whereas dietary self-control engages prefrontal processes that enhance the influence of health information on value perceptions [57,58]. Food images may therefore compete more successfully for motivated attention than abstract calorie information (though this is beyond the data presented here, as the present study did not directly measure neural activation or executive control).

This pattern of visual switching also aligns with the literature on approach–avoidance conflict in eating disorders [59]. Rather than food cues purely signaling reward, they can also represent threats to eating goals, which might explain the higher number of fixations without a corresponding increase in the total duration. This makes more focused deliberation less stable but active engagement with the visual cue more prominent. Non-restricted eaters may be switching between food cues as much as restricted eaters are switching between food cues and calorie labels. Studies examining gaze tracking should consider this possibility in future research.

Several limitations of this study should be noted. First, this study only examined the visual attention paid to food cues and calorie labels and not the actual consumption choices. While it is assumed to be important to the processing of decision making, visual attention is not a consumption decision and may have a weak association with the direction of consumption decisions. Future work should examine whether the observed attentional patterns predict choices and consumption in real-world contexts. Further, the decision-making context used in this study also warrants consideration. Participants were asked to select items they would typically choose for a meal at a fast-food restaurant. However, decision making may differ across types of individuals and contexts, such as routine meals versus indulgent or “treat” occasions. The influence of visual cues may vary accordingly, and future research should explore these contextual differences.

Similarly, multiple concerns exist regarding ecological validity. The use of simplified, non-naturalistic menus that excluded factors such as pricing, promotions, sizing, branding, and additional nutritional information allowed for experimental control but limited ecological validity. In real-world settings, these elements interact with visual cues and informational content in complex ways and may significantly influence attention and ultimate decision making. Future studies should incorporate more comprehensive and realistic menu environments to better understand how these factors jointly shape attentional focus and food choices.

Lastly, the sample and research design deserve some consideration. The sample consisted exclusively of young adults. Although this group represents a primary consumer base for fast food, their eating motivations and responsiveness to food cues may differ from those of the general population. Younger individuals may also be more reactive to appetitive cues, potentially amplifying the effects observed here. Further, our sample was predominantly female, and individuals who identify as female also report seeing and using calorie labels more often than males [11]. In addition to these differences, restricted eaters represented a small number of people in this sample, even though we adopted a very broad operationalization of restriction. This limits generalizability of these findings statistically as well as conceptually but offers interesting insights for future research.

## 5. Conclusions

Taken together, the results of this study provide strong evidence for an attentional asymmetry in food decision-making environments, which fits with both existing theory and previous empirical data. Less motivationally relevant cues such as calorie labels increase attention (time spent looking and deciding) in a parallel, additive manner, whereas appetitive food cues both attract more attention in general and distract from competing information like calorie labels. This asymmetry suggests that visual attention is heavily influenced by the motivational relevance of food cues exerting a dominant influence over attention and informational processing. Enhancing the motivational relevance of calorie and related information, alongside a deeper understanding of contextual and individual factors influencing motivated attention, is necessary to improve the effectiveness of calorie labeling interventions.

## Figures and Tables

**Table 1:** Menu item energy density across array and exemplar restaurants.

Higher Energy Density				Lower Energy Density			
Item	kcal	g	ED	Item	kcal	g	ED
Chick-fil-A							
Chicken sandwich	440	183	2.40	Side salad	160	166	0.96
Deluxe spicy chicken sandwich	550	259	2.12	Grilled chicken nuggets	185	95	1.95
Deluxe chicken sandwich	500	247	2.02	Yogurt parfait	250	221	1.13
Spicy chicken sandwich	460	188	2.45	Grilled chicken sandwich	310	206	1.50
Waffle fries	360	125	2.88	Market salad	440	321	1.37
Chicken strips	375	136	2.76	Grilled chicken wrap	360	231	1.56
Burger King							
Rodeo King cheeseburger	1250	411	3.04	MorningStar veggie burger	320	198	1.62
Bacon King cheeseburger	1150	362	3.18	Whopper Jr. sandwich	310	142	2.18
Bacon cheese chicken sandwich	1340	430	3.12	Grilled chicken sandwich	470	240	1.96
Double Whopper cheeseburger	980	372	2.63	Chicken club salad	540	380	1.42
Cheesy tots	316	130	2.43	Garden side salad	40	99	0.40
French fries	321	160	2.01	Chicken garden salad	320	308	1.04
Taco Bell							
Chalupa supreme	370	153	2.42	Chips and salsa	180	106	1.70
Burrito supreme	500	240	2.08	Black beans	80	62	1.29
XXL Grilled Stuft burrito	870	387	2.25	Chicken soft taco	170	92	1.85
Nachos Belgrande	750	308	2.44	Double decker taco	350	184	1.90
Crunchwrap supreme	540	265	2.04	Crunchy taco	170	113	1.50
Quesorito	650	259	2.51	Black bean burrito	380	204	1.86
Arby’s							
Curly fries	240	77	3.12	Side salad	70	133	0.53
Greek gyro	710	273	2.60	Turkey farmhouse salad	240	304	0.79
Mozzarella sticks	440	137	3.21	Roast beef sandwich	540	327	1.65
Turkey bacon ranch sandwich	810	344	2.35	Roast beef slider	180	84	2.14
Chicken Cordon Bleu sandwich	660	268	2.46	Crispy chicken salad	430	334	1.29
Apple turnover	430	128	2.40	Jalapeno bites	290	176	1.65

**Table 2:** Influences of calorie label presence and individual differences on number of fixations.

	Calorie Labels										Food Cues									
	Null					Full Model/Final Model					Null					Full Model/Final Model				
Fixed Effect	*B*	S.E.	*t*	*df*	*p*	*B*	S.E.	*t*	*df*	*p*	*B*	S.E.	*t*	*df*	*p*	*B*	S.E.	*t*	*df*	*p*
Intercept	15.92	1.81	8.81	81	<0.001	−0.57	3.79	−0.15	73	0.88	27.30	2.88	9.47	81	<0.001	25.24	8.74	2.89	73	0.005
Gender						0.56	2.61	0.22	73	0.83						1.09	6.01	0.18	73	0.86
Age						1.59	1.51	1.05	73	0.30						−0.56	1.98	−0.28	73	0.78
Race						−1.06	1.38	−0.77	73	0.45						−0.50	2.33	−0.21	73	0.83
Hours						−0.12	0.32	−0.38	73	0.71						−0.14	0.58	−0.25	73	0.81
Hungry						−0.50	1.10	−0.45	73	0.65						1.79	1.98	0.91	73	0.37
PSS10						−2.73	2.86	−0.96	73	0.34						−6.29	4.76	−1.32	73	0.19
Restrict						11.30	4.84	2.33	73	0.02						7.94	6.74	1.18	73	0.24
BMI						0.05	0.41	0.12	73	0.91						0.21	0.76	0.28	73	0.78
Cal Label						30.38	3.49	8.69	81	<0.001						−0.28	2.55	−0.11	81	0.91
ICC	0.001 (NS)										0.76 (S)									
	Null					Full Model/Final Model					Null					Full Model/Final Model				
Random Effects	VC	S.D.	*df*	χ^2^	*p*	VC	S.D.	*df*	χ^2^	*p*	VC	S.D.	*df*	χ^2^	*p*	VC	S.D	*df*	χ^2^	*p*
WS Variance	689.32	26.25				424.54	20.60				188.10	13.72				190.20	13.79			
BS Variance	0.70	0.84	81	63.66	>0.50	29.01	5.39	73	82.71	0.21	595.59	24.40	81	593.94	<0.001	635.34	25.21	73	560.35	<0.001

**Table 3:** Influences of calorie label presence and individual differences on duration of fixations.

	On Calorie Labels										On Food Cues									
	Null					Full Model/Final Model					Null				Full Model/Final Model					
Fixed Effect	*B*	S.E.	*t*	*df*	*p*	*B*	S.E.	*t*	*df*	*p*	*B*	S.E.	*t*	*df*	*p*	*B*	S.E.	*t*	*df*	*p*
Intercept	5.78	0.77	7.49	81	<0.001	−0.49	1.55	−0.32	73	0.75	7.62	0.86	8.87	81	<0.001	6.36	2.62	2.43	73	0.02
Gender						0.44	1.03	0.43	73	0.67						1.01	1.76	0.58	73	0.57
Age						0.78	0.66	1.19	73	0.24						−0.31	0.57	−0.54	73	0.59
Race						−0.44	0.54	−0.82	73	0.41						−0.31	0.65	−0.48	73	0.63
Hours						−0.05	0.14	−0.39	73	0.70						−0.11	0.16	−0.69	73	0.49
Hungry						−0.36	0.52	−0.69	73	0.49						0.73	0.59	1.25	73	0.22
PSS10						−1.82	1.44	−1.27	73	0.21						−1.67	1.51	−1.11	73	0.27
Restrict						3.44	2.11	1.63	73	0.11						1.81	1.94	0.93	73	0.36
BMI						<0.001	0.16	0.002	73	1.00						0.09	0.22	0.39	73	0.70
Cal Label						11.26	1.53	7.36.	81	<0.001						−0.20	0.85	−0.24	81	0.81
ICC	0.001 (NS)										0.72 (S)									
	Null					Full Model/Final Model					Null				Full Model/Final Model					
Random Effects	VC	S.D.	*df*	χ^2^	*p*	VC	S.D.	*df*	χ^2^	*p*	VC	S.D.	*df*	χ^2^	*p*	VC	S.D	*df*	χ^2^	*p*
WS Variance	118.43	10.88				81.63	9.04				20.04	4.48				20.24	4.50			
BS Variance	0.14	0.37	81	67.60	>0.50	4.90	2.21	73	81.41	0.23	51.38	7.17	81	496.33	<0.001	54.79	7.40	73	468.00	<0.001

**Table 4:** Influences of food cue presence and individual differences on number of fixations.

	Calorie Labels										Food Cues									
	Null					Full Model/Final Model					Null					Full Model/Final Model				
Fixed Effect	*B*	S.E.	*t*	*df*	*p*	*B*	S.E.	*t*	*df*	*p*	*B*	S.E.	*t*	*df*	*p*	*B*	S.E.	*t*	*df*	*p*
Intercept	16.02	1.66	9.7	81	<0.001	23.82	10.39	2.29	73	0.03	27.74	3.16	8.79	81	<0.001	13.79	19.96	0.69	73	0.49
Gender						1.57	3.35	0.47	73	0.64						−9.03	5.85	−1.54	73	0.13
Age						−1.87	1.35	−1.39	73	0.17						−0.57	2.19	−0.26	73	0.80
Race						−0.60	1.05	−0.58	73	0.57						0.21	2.23	0.09	73	0.93
Hours						0.21	0.29	0.74	73	0.46						0.53	0.49	1.07	73	0.29
Hungry						1.49	1.05	1.43	73	0.16						1.80	2.12	0.85	73	0.40
PSS10						−0.86	2.59	−0.33	73	0.74						3.94	5.95	0.66	73	0.51
Restrict						5.38	5.55	0.97	73	0.34						13.33	9.46	1.41	73	0.16
BMI						−0.25	0.35	−0.72	73	0.48						−0.02	0.76	−0.02	73	0.98
Food Cue						−8.3	2.27	−3.66	81	<0.001						48.25	6.58	7.33	81	<0.001
ICC	0.44 (S)										0.001 (NS)									
	Null					Full Model/Final Model					Null					Full Model/Final Model				
Random Effects	VC	S.D.	*df*	χ^2^	*p*	VC	S.D.	*df*	χ^2^	*p*	VC	S.D.	*df*	χ^2^	*p*	VC	S.D.	*df*	χ^2^	*p*
WS Variance	177.70	13.3				151.66	12.32				2064.95	45.44				1350.92	36.75			
BS Variance	139.01	11.8	81	207.73	<0.001	151.70	12.32	73	218.66	<0.001	2.38	1.54	81	64.89	>0.50	232.46	15.24	73	97.78	0.03

**Table 5:** Influences of food cue presence and individual differences on duration of fixations.

	Calorie Labels										Food Cues									
	Null					Full Model/Final Model					Null					Full Model/Final Model				
Fixed Effect	*B*	S.E.	*t*	*df*	*p*	*B*	S.E.	*t*	*df*	*p*	*B*	S.E.	*t*	*df*	*p*	*B*	S.E.	*t*	*df*	*p*
Intercept	5.83	0.72	8.11	81	<0.001	11.13	4.53	2.45	73	0.02	7.74	0.93	8.30	81	<0.001	2.89	5.80	0.50	73	0.62
Gender						0.28	1.30	0.21	73	0.83						−2.41	1.52	−1.59	73	0.12
Age						−0.83	0.61	−1.37	73	0.17						−0.30	0.67	−0.44	73	0.66
Race						−0.07	0.49	−0.13	73	0.90						−0.09	0.57	−0.16	73	0.87
Hours						0.12	0.13	0.87	73	0.39						0.12	0.14	0.85	73	0.40
Hungry						0.34	0.43	0.79	73	0.43						0.41	0.61	0.67	73	0.51
PSS10						−1.45	1.02	−1.42	73	0.16						1.08	1.78	0.61	73	0.55
Restrict						2.61	2.34	1.11	73	0.27						4.94	2.96	1.67	73	0.10
BMI						−0.16	0.16	−1.03	73	0.31						0.04	0.22	0.17	73	0.87
Food Cue						−4.36	1.07	−4.09	81	<0.001						13.03	1.99	6.55	81	<0.001
ICC	0.35 (S)										0.001 (NS)									
	Null					Full Model/Final Model					Null					Full Model/Final Model				
Random Effects	VC	S.D.	*df*	χ^2^	*p*	VC	S.D.	*df*	χ^2^	*p*	VC	S.D.	*df*	χ^2^	*p*	VC	S.D	*df*	χ^2^	*p*
WS Variance	41.29	6.43				33.72	5.81				179.95	13.41				130.17	11.41			
BS Variance	22.19	4.71	81	168.07	<0.001	26.00	5.10	73	185.27	<0.001	0.18	0.43	81	65.14	>0.50	12.92	3.59	73	87.44	0.12

## Data Availability

The original contributions presented in this study are included in the article/Supplementary Materials. Further inquiries can be directed to the corresponding author.

## Supplementary Materials

The following supporting information can be downloaded at: https://www.mdpi.com/article/10.3390/nu18162620/s1, Table S1: Summary of Measures Collected and Their Inclusion in the Present Manuscript.
