# Supplementary material for: Eyes on the Fries: An Eye-Tracking Study of Motivated Attention and Calorie Labeling on Fast-Food Menus
Source: Nutrients. 2026 Aug 11;18(16):2620. doi: 10.3390/nu18162620 (PMC13516153; doi:10.3390/nu18162620)
Supplement: Supplementary file 1 [file nutrients-18-02620-s001.zip › nutrients-4432411-supplementary.pdf]

Table S1
Summary of Measures Collected and Their Inclusion in the Present Manuscript

Measure	Construct Assessed	Time Point	Role in Current Study	Reported in the Present Manuscript
Visual attention (fixation count)	Selective visual attention	During stimulus	Primary theoretical outcome	Yes
Visual attention (fixation duration)	Sustained visual attention	During stimulus	Primary theoretical outcome	Yes
Restrained eating	Individual difference	Baseline	Moderator	Yes
Food selection	Behavioral choice	Post-stimulus	Behavioral outcome	Planned future report
Heart rate	Cognitive resource allocation	During stimulus	Psychophysiological outcome	Planned future report
Skin conductance	Physiological arousal	During stimulus	Psychophysiological outcome	Planned future report
Facial electromyography (i.e., corrugator supercilii, orbicularis oculi, zygomaticus major, levator labii)	Emotional valence	During stimulus	Psychophysiological outcome	Planned future report
Self-report measures (e.g., hunger, demographics)	Participant characteristics	Baseline	Covariates	As applicable

Note. This table summarizes all measures collected as part of the study. The present manuscript focuses on testing hypotheses derived from the motivated attention framework, with eye-tracking measures serving as the primary theoretical outcomes. Additional behavioral and psychophysiological measures were collected to address separate research questions and are planned for future reports.
